# Long-Term Effects of Subacute Ruminal Acidosis (SARA) on Milk Quality and Hepatic Gene Expression in Lactating Goats Fed a High-Concentrate Diet

**DOI:** 10.1371/journal.pone.0082850

**Published:** 2013-12-23

**Authors:** Haibo Dong, Shaoqing Wang, Yuanyuan Jia, Yingdong Ni, Yuanshu Zhang, Su Zhuang, Xiangzhen Shen, Ruqian Zhao

**Affiliations:** 1 Key Laboratory of Animal Physiology & Biochemistry, Ministry of Agriculture, Nanjing Agricultural University, Nanjing, Jiangsu, China; 2 College of Animal Sciences and Technology, Nanjing Agricultural University, Nanjing, Jiangsu, China; 3 Institute of Small Animal Disease, College of Veterinary Medicine, Nanjing Agricultural University, Nanjing, Jiangsu, China; University of Illinois, United States of America

## Abstract

**Purpose:**

The mechanism underlying the decline in milk quality during periods of feeding high-concentrate diets to dairy ruminants is not well documented. The aim of this study was to investigate the metabolic changes in the liver that contribute to the input of substrate precursors to the mammary gland after feeding a high-concentrate diet to lactating goats for a long period.

**Experimental Design:**

Eight mid-lactating goats with rumen cannulas were randomly assigned to two groups. For 9 weeks, the treatment group was fed a high-concentrate diet (60% concentrate of dry matter, HC) and the control group was fed a low-concentrate diet (40% concentrate of dry matter, LC). Ruminal fluid, plasma, and liver tissues were sampled, microarray techniques and real-time polymerase chain reaction were used to evaluate metabolic parameters and gene expression in liver.

**Results:**

Feeding a 60%-concentrate diet for 9 weeks resulted in a significant decrease in rumen pH. Changes in fat and protein content also occurred, which negatively affected milk quality. Plasma levels of leptin (*p = 0.058*), non-esterified fatty acid (*p = 0.071*), and glucose (*p = 0.014*) increased markedly in HC group. Plasma cortisol concentration was significantly elevated in the treatment group (*p<0.05*). Expression of the glucocorticoid receptor protein gene was significantly down-regulated (*p<0.05*) in the liver. The expression of genes for interleukin 1β, serum amyloid A, C-reactive protein, and haptoglobin mRNA was significantly increased (*p<0.05*) in the HC group. GeneRelNet analysis showed that gene expression involved in inflammatory responses and the metabolism of lipids, protein, and carbohydrate were significantly altered by feeding a high-concentrate diet for 9 weeks.

**Conclusions:**

Activation of the acute phase response and the inflammatory response may contribute to nutrient partitioning and re-distribution of energy in the liver, and ultimately lead to a decline in milk quality.

## Introduction

Dairy ruminants are often fed a high-concentrate diet to meet the energy requirements of high milk production. However, metabolic disorders such as subacute ruminal acidosis (SARA) can result from feeding excessive amounts of high proportions of fermentable concentrate and forage with low physically effective fiber [1]. SARA is a common digestive disorder in most dairy herds. In intensive management systems, 19% of early lactation and 26% of mid-lactation cows can be affected by SARA [2]. The consequences of SARA include depression of food intake, diarrhea, laminitis, and inflammatory response, which ultimately result in the depression of milk quality and quantity [3] [4] [5] [6]. Production costs from SARA have been estimated to be as high as $1.12 per cow per day [7]. SARA is a major concern for the global dairy industry. Especially, in the regions shortage of high quality forage and lack of reasonable feeding management practices prevents dairy cows from achieving their genetic potential for milk production.

SARA is experimentally characterized by repeated bouts of depressed ruminal pH values between 5.2 and 5.6 for at least 3 hours per day or below5.8 [5] [6]. Overfeeding fast fermentable starch or feeding excessive concentrate to forage ratios for 2 to 3 weeks can induce SARA clinical signs [2]. Most studies have focused on the short-term (2–3 weeks) effects of SARA on inflammatory response, rumen fermentation, and milk production. Fairfield et al. [7] and Khafipoor [8] reported that experimentally induced SARA decreased milk fat percentage but increased milk protein content. However, other reports have suggested that during the short term SARA did not affect milk fat or protein content [9] [10]. However, dairy production herds affected by SARA can experience negative outcomes as long as several weeks (>3 weeks) after an episode [11]. These diseases include laminitis, weight loss, and unexplained abscesses. The long-term metabolic effects of SARA have not been well studied.

Feeding high-concentrate diets to lactating cows results in the release of bacterial endotoxins, such as lipopolysaccharide (LPS), from the rumen or hind gut [12]. Free LPS can translocate into the bloodstream from the digestive tract under conditions of high permeability and after injury to the epithelial tissue [12]. Immune responses are then activated and pro-inflammatory cytokines are released from toll-like receptors 4 (TLR-4) that recognize the circulating LPS [13]. The process of LPS recognition via TLR-4 is facilitated by the accessory molecule LPS-binding protein (LBP) and cluster of differentiation antigen 14 (CD14) [14] [15]. LBP is an acute phase protein (APP). The acute phase response to infection, tissue injuries, or other disorders participates in the restoration of physiological homeostasis [16]. The liver is the major site for the synthesis of APPs, including LBP, C-reactive protein (CRP), serum amyloid A (SAA), and haptoglobin (HP) [17] [18] [19]. Monokines such as interleukin 6 (IL-6), IL-1, and tumor necrosis factor alpha (TNF-α) induce APP synthesis via their specific hepatic receptors [20] These monokines also stimulate the release of adrenocorticotropic hormone (ACTH) from the pituitary gland. ACTH signals an increased secretion of glucocorticoid hormone from the adrenal cortex [21]. During the restoration of physiological homeostasis, these mediators act on specific receptors on different target cells, which results in changes in metabolism and systemic responses.

As the vital organ that controls metabolism [22], the liver is stimulated by endogenous and exogenous factors. It is responsible for nutrient partitioning, including homeostatic and long-term homeorhetic adaptations [23]. Physical and psychological stress elevates plasma IL-6 and APP levels in humans and in experimental animals [24] [25]. Although the mechanism of APP induction in response to stress is yet to be elucidated, activation of the hypothalamic–pituitary–adrenal (HPA) axis by stressors may trigger systemic or local cytokine production. It's reported that, during stress response physiological processes are aimed on redistribution of energy utilization in specific organs, inhibiting or stimulating energy mobilization according to priority [26]. As a fact, defense against infectious diseases and stress response are resource expensive [27]. Therefore, the objective of this study was to investigate the changes of acute phase response, stress and inflammatory response, as well as the metabolic changes in liver that may contribute to the input of substrate precursors to the mammary gland after feeding a high-concentrate diet to lactating goats for a long period.

## Results

### Ruminal fluid pH

**Figure 1:**
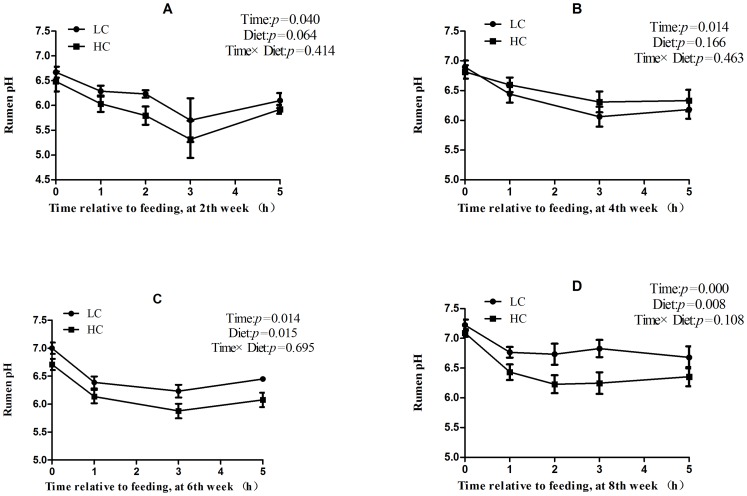
Rumen fluid pH values. Data were analyzed for differences due to diet, time, and their interactions by Univariate using the General Linear Models of SPSS 11.0 for Windows (StatSoft Inc, Tulsa, OK, USA). Values are mean ± standard error of the mean (SEM).

After 1 week of feeding a high-concentrate diet, the mean ruminal pH value in the HC group was pH <5.8 from 1 to 5 hours after feeding, which was consistent with the experimental definition of SARA *(* *figure 1A* *)*. During week 4 to week 8, enhanced ruminal buffering capacity and diet adaptation resulted in an increase in pH of HC group (mean pH >6.0), but it was still lower than that of LC group *(* *figure 1B, C and D* *)*.

### Plasma biochemical parameters and milk composition

In HC goats compared with LC counterparts, the levels of plasma non-esterified fatty acid (NEFA; *p = 0.071*) and leptin (*p = 0.058*) had a tendency to increase and blood glucose (*p = 0.014*) was significantly increased after feeding high concentrate diet for 9 weeks (Table 1).

**Table 1:** Comparison of plasma concentrations of glucose, non-esterified fatty acids (NEFA), triglyceride (TG) and leptin.

Diet			
Parameters	LC	HC	*P*-value
Glucose	3.00±0.06	3.3±0.05	0.014
NEFA (μmol/L)	175.87±55.12	969.52±293.20	0.071
TG (mmol/L)	0.19±0.06	0.18±0.03	0.659
Leptin (ng/mL)	1.75±0.57	2.01±0.78	0.058

LC, low concentrate; HC, high concentrate; NEFA, non-esterified fatty acid. TG, triglyceride. Values are mean ± standard error of the mean (SEM), *n* = 4/group.

The percentage of milk fat and protein decreased significantly from 5–9 weeks in HC group compared with LC group (*p<0.05*). However, milk lactose concentration increased significantly in HC group from the 4th week of treatment compared to LC group (*p<0.05*; Table 2).

**Table 2:** The percentage of fat, protein, and lactose in milk from control and treatment group goats.

Item	Diet	Time				Effect, *P*-value		
		0–2 W	3–4 W	5–6 W	7–9 W	Diet	Time	Diet × Time
Fat	LC	4.15±0.43	4.01±0.38	3.76±0.88	3.90±0.53			
	HC	3.09±0.63*	3.34±1.15	3.20±1.22*	3.04±0.89**	0.931	0.021	0.935
Protein	LC	2.90±0.35	3.26±0.16	3.10±0.67	3.18±0.10			
	HC	2.88±0.28	2.92±0.40	2.62±0.41*	2.68±0.22**	0.516	0.007	0.372
Lactose	LC	4.82±0.23	4.59±0.33	4.72±0.28	4.78±0.29			
	HC	4.90±0.28	4.96±0.09*	5.00±0.10	4.95±0.13*	0.845	0.017	0.646

LC, low concentrate; HC, high concentrate; Values are mean ± standard error of the mean (SEM), *n* = 4/group. * p<0.05, ** P<0.01 vs. LC.

### LPS content in ruminal fluid and plasma

LPS levels in ruminal fluids of HC group increased from 38,854 to 48,064 EU/mL before feeding in the morning and increased from 63,359 to 70,418 EU/mL after 8 h feeding compared to LC group. Plasma LPS concentration of HC goats was increased from 0.24 to 0.32 EU/mL before feeding in the morning and from 0.09 to 0.14 EU/mL after 4 h feeding compared with LC group. However, there was no significant difference in plasma LPS concentration and ruminal LPS content between HC and LC group (*p>0.05*; Table 3).

**Table 3:** Lipopolysaccharide (LPS) levels in plasma and in rumen fluid of control and treatment group goats.

Items	Diet		SEM	Effect, *P*-value		
	LC	HC		Diet	Time	Diet × Time
Plasma LPS, EU/mL						
0h	0.24	0.32	0.05	0.249	0.002	0.716
4h	0.09	0.14				
8h	0.07	0.06				
Rumen LPS^1^, EU/mL						
0h	38,854	48,064	20,383	0.717	0.084	0.98
4h	14,466	16,058				
8h	63,359	70,418				

LC, low concentrate; HC, high concentrate; LPS, lipopolysaccharide; Values are mean ± standard error of the mean between two groups (SEM), *n* = 4/group.

^1^ Statistical analysis conducted on log_10_−transformd date.

### Cytokines, acute phase proteins

Plasma concentrations of IL-6 and TNF-α increased in HC group but were not significantly different compared to the LC group (*p>0.05*; Table 4). Compared with LC group, plasma SAA concentration in HC group was increased significantly (*p<0.05*), and plasma HP concentration also showed a tendency to increase (*p = 0.08*). Results for plasma LBP were not significantly different for the HC group compared to the LC group (*p = 0.163*).

**Table 4:** Plasma cytokines and acute phase protein levels in control and treatment group goats.

	Diet		
Parameters	LC	HC	*P*-value
IL-6(μg/mL)	221.32±42.63	345.45±56.33	0.163
TNF-α(ng/mL)	715.42±49.67	984.36±130.46	0.126
SAA(μg/mL)	90.62±15.67	246.04±37.37	0.020
HP(μg/mL)	209.50±21.99	307.54±35.77	0.087
LBP(ng/mL)	23.32±6.10	34.48±5.74	0.163

LC, low concentrate; HC, high concentrate; HP, haptoglobin; IL-6, interleukin-6; LBP, lipopolysaccharide binding protein; SAA, serum amyloid A protein; TNF-α, tumour necrosises factor α. Values are mean ± standard error of the mean (SEM), *n* = 4/group.

### Cortisol and glucocorticoid receptor

**Figure 2:**
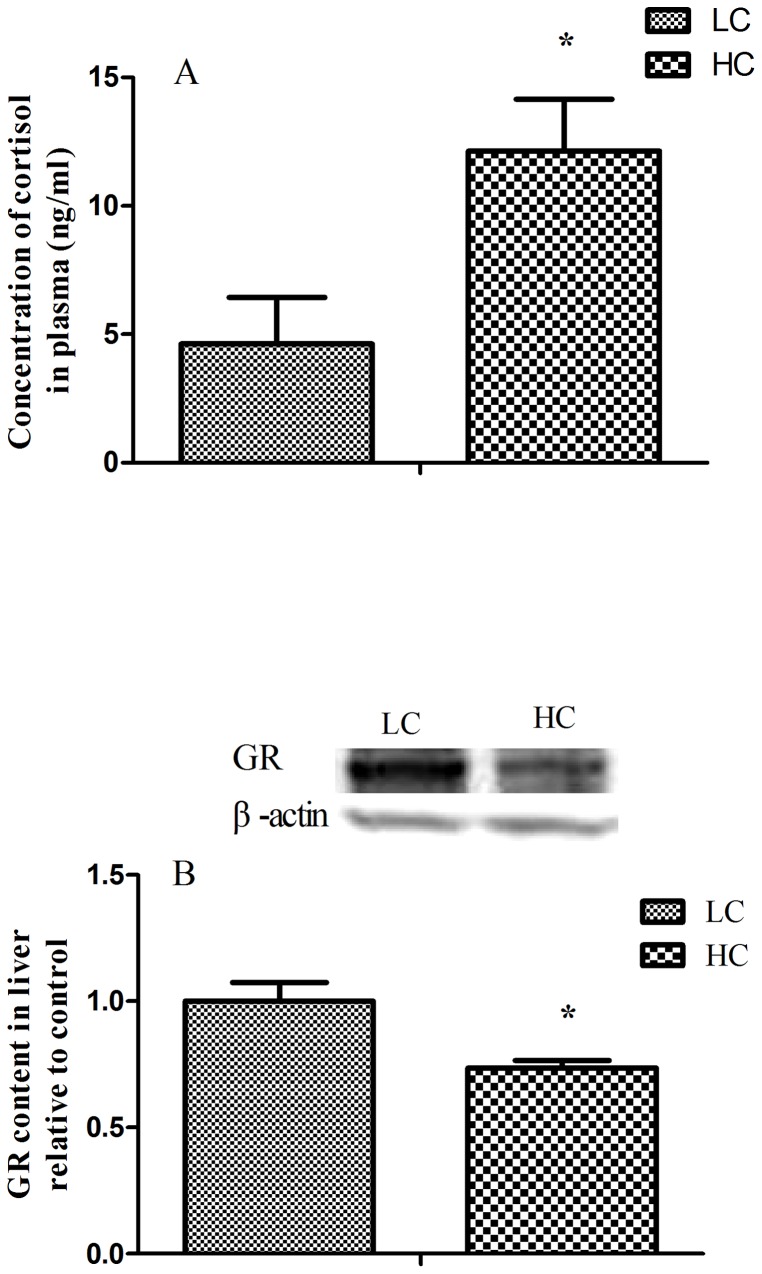
Plasma cortisol concentration (A) and hepatic glucocorticoid receptor (GR) protein expression (B). The concentrations of cortisol were significantly up-regulation and the GR protein expression was significantly down-regulation. Values are mean ± standard error of the mean (SEM); *p<0.05 vs. LC.

The level of plasma cortisol was significantly elevated in HC group (*p<0.05*). Glucocorticoid receptor (GR) protein expression in the liver was greatly down-regulated in HC group compared with LC group (*p<0.05*; *figure 2*).

### Liver mRNA Expression Profiling by Microarray

**Figure 3:**
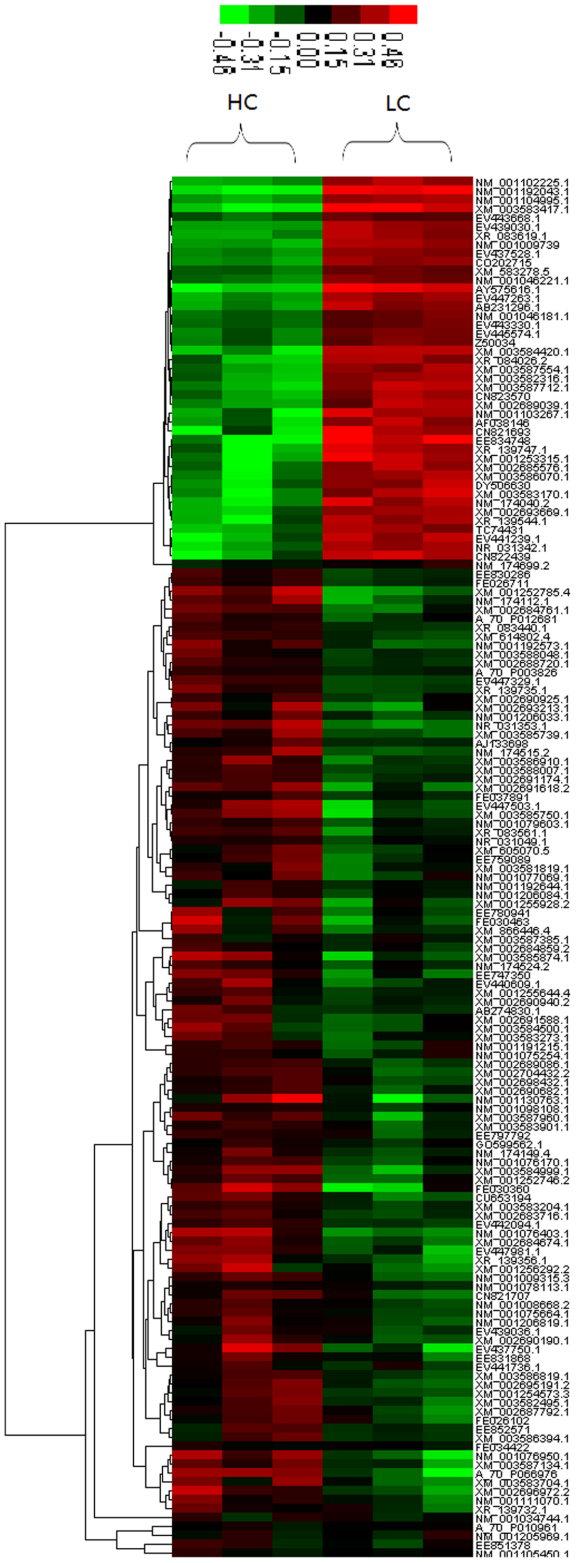
Hierarchical cluster analysis of the differentially expressed transcripts (n = 155). 155 transcripts were differentially expressed (adjusted *p<0.01*). Red and green colors reflect the high and low intensities, respectively.

Approximately 512 genes were observed to be differentially expressed in the HC group with approximately two-thirds of the genes exhibiting increased expression in liver. After removal of redundant and unannotated sequences (*p<0.01*), 43 genes were significantly down-regulated and 112 genes were significantly up-regulated in treated goats compared to LC group (*figure 3*). Functional annotation of enriched Gene Ontology terms indicates that a number of biological processes within the liver are affected by consumption of high-concentrate diets, including those related to control of cell surface receptor linked signal-transduction, G-protein coupled receptor protein signaling, regulation of transcription, immune response, and proteolysis.

The raw data and processed files for the two libraries have been deposited in NCBI's Gene Expression Omnibus database (http://www.ncbi.nlm.nih.gov/geo/) under accession number GPL17623 and GPL17624.

### The GeneRelNet (Co-expression network) analysis

**Figure 4:**
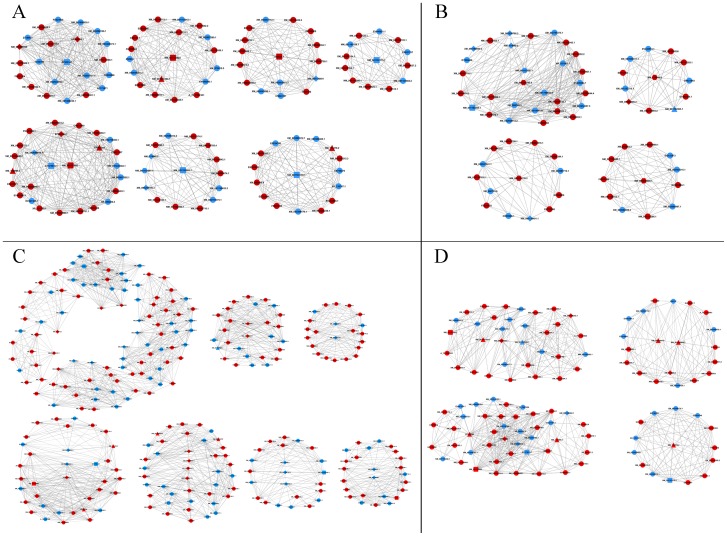
Co-expression network for k-core genes in liver of HC goats. Co-expression networks were for immune and inflammatory responses (A), lipid metabolism (B), protein (or amino acid) metabolism (C), and carbohydrate metabolism (D) in the liver of HC goats. Circles in the figure represent genes (red circle means up–regulation; blue circle means down-regulation), lines represent regulation relationships between genes (solid lines mean a positive correlation of the expression between genes; dotted lines mean a negative correlation), rectangles represent genes for immunity and stress, triangles represent carbohydrate metabolism, hexagons represent lipid metabolism, and diamonds represent protein metabolism.

Co-expression network analysis was used to find K-core regulatory factors (genes) (*figure 4A–D*). For each pair of genes in a cluster, we calculated the Pearson correlation coefficient and chose the significant correlation pairs to construct the network (absolute value of interaction >0.999). These genes were mainly involved in immune and inflammatory responses (n = 8), lipid metabolism (n = 5), protein metabolism (n = 26), carbohydrate metabolism (n = 7), out of a total of 155 differential expression genes (*p<0.01*).

### Real-time polymerase chain reaction (PCR) analysis involved in inflammatory response, glucose metabolism and lipid metabolism

**Figure 5:**
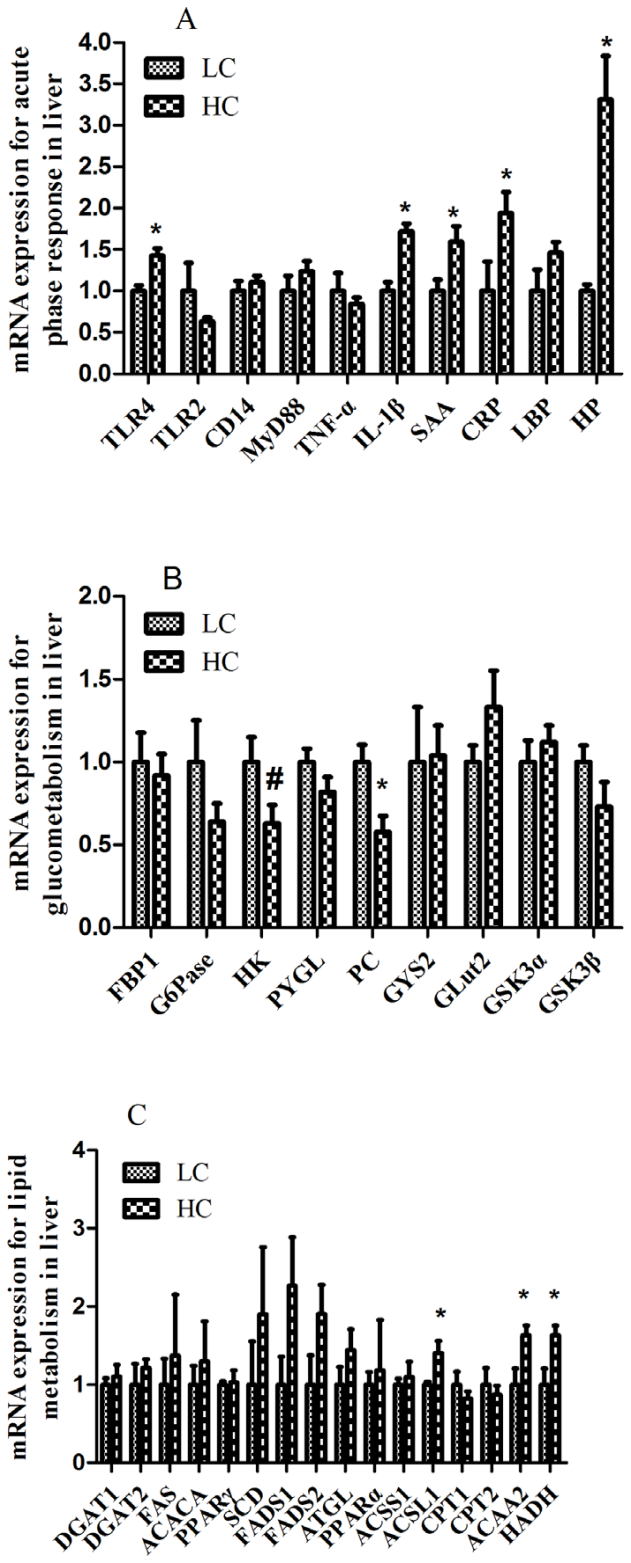
Hepatic gene expression profile detected by real-time PCR. (A) mRNA expression of the key genes for inflammatory response and acute phase responses. (B) mRNA expression of the key genes for glucometabolism. (C) mRNA expression of the key genes for lipid metabolism. Beta-actin was used as the reference gene for gene expression. Values are mean ± SEM. *^#^p<0.1*, *p<0.05 vs. LC.

The mRNA expression of hepatic TLR-4, IL-1β, SAA, CRP, and HP were significantly increased in HC group compared to LC counterparts (*p<0.05*; *figure 5A*). The expressions of hepatic TLR2, CD14, MyD88, TNF-α, and LBP did not show significant difference between HC and LC group (*p*>*0.05*). Among these genes, only HP mRNA expression showed a more than 2-fold difference between the two groups, which was consistent with the data from the transcriptome microarray.

Expression of hepatic phosphoenolpyruvate carboxykinase (PC) mRNA was significantly down-regulated in HC group compared to LC group (*p<0.05*) (*figure 5B*). Hexokinase (HK) mRNA expression had a tendency to decrease in HC group (*p = 0.07*). However, other gluconeogenesis- and glucolysis-related genes transcription was not significantly altered in HC group compared to LC group (*p*>*0.05*).

The mRNA expression of hepatic acetyl-CoA acyltransferase 2 (ACAA2), acyl-CoA synthetase long-chain family member-1 (ACSL1), and hydroxyacyl-CoA dehydrogenase (HADH) genes were significantly increased (*p<0.05*) in HC group compared to LC group (*figure 5C*). There was a >2-fold difference in hepatic fatty acid desaturase 1 (FADS1) mRNA expression, but did not reach statistical significance due to the high variability between individual values. This result was consistent with the data from the microarray high-throughput data analysis.

## Discussion

### Dynamic changes in ruminal pH during SARA

Dairy ruminants are often fed high grain diets to meet the energy demand for high milk production. As a result, ruminal acidosis (especially SARA) often occurs during the periods of early and mid-lactation in dairy production herds [13] [11]. Due to the rapid fermentation and the accumulation of volatile fatty acid (VFA) in the rumen, as well as the increase of lactic acid, the value of rumen pH markedly decreased and prolonged for long time [28] [29]. In experimental study, rumen pH value has been considered as the most effective indicator of SARA. The thresholds of ruminal pH for the diagnosis of SARA have been set as ruminal pH <5.6 [10] [30] [31] and <5.8 [28] lasted for 3 hours per day using ruminal fluids detected by pH meter system in dairy cows [10]. Consistently, ruminal pH value <5.8 lasted more than 3 hours was considered as the critical value of SARA diagnosis [32].

In this study, goats fed a high-concentrate diet for 2 weeks exhibited a significantly lower ruminal pH, which decreased to <5.8 and persisted for more than 3 hours per day after feeding. According to the definition of experimental SARA, HC goats were suffered SARA disease. However, although ruminal pH value of HC goats remained significantly lower than LC goats from week 4 (4W) to week 8 (8W), the ruminal pH in HC goats was markedly increased to more than 6.0. The increase in ruminal pH of HC goats indicated that the rumen was adapting to the high-concentrate diet, which was consistent with the study conducted in dairy cows by Steele et al [33].

### Inflammatory response and HPA axis activation

It is widely accepted that during experimentally-induced SARA, the free LPS contents were increased in rumen fluids, yet LPS concentration in blood was vague after feeding HC diet that has been enriched with starch [15]. A decline in ruminal pH during SARA causes death and cell lysis of Gram-negative bacteria, the predominant bacterial group in the rumen, which results in an increase in free ruminal LPS concentration [12] [15], [17]. Endotoxin produced in the digestive tract can be translocated into the bloodstream, which increases the concentration of blood LPS [5] [7]. It's generally accepted that LPS translocation into the bloodstream occurs as a consequence of grain-induced SARA, and that the intestines are likely the main site of LPS translocation [15] [16]. In grain-based SARA, more starch may flow into the hind gut especially into the large intestine where LPS may be produced in a significant amount as in the rumen via microorganism fermentation [9]. After LPS translocates into the peripheral circulatory system, the inflammatory and acute phase response will be activated [12]. Our results showed that, LPS concentrations were increased from 38,854 to 48,064 EU/mL before feeding in the morning and increased from 63,359 to 70,418 EU/mL after 8h feeding compared to LC group. Plasma LPS concentration of HC goats was increased from 0.24 to 0.32 EU/mL before feeding in the morning and from 0.09 to 0.14 EU/mL after 4 h feeding compared with LC group. However, due to the limited animal sizes and the individual variations the difference of LPS concentrations in ruminal fluids and plasma between HC and LC goats did not reach the statistical significance, it can't be discarded whether this moderate increase of LPS in body fluids involved in activation of inflammatory response. The activation of TLR4 and IL-1β mRNA expression in liver of HC goats also supported the above speculation. Compared to LC group, HC goats showed a significant up-regulation in mRNA expression of APPs, including hepatic SAA, CRP, and HP mRNA, and paralleled a marked increase of SAA and HP in the plasma. These results were similar to results reported in dairy cattle [7] [13] [15].

The previous studies showed that the stress axis is activated when SARA is induced [34]. Cytokines and APPs are strong inducers that signal the HPA axis to release glucocorticoids from the adrenal gland [11] [35] [36]. In the present study, we found that goats fed a high-concentrate diet for 9 weeks had a significantly higher concentration of peripheral blood cortisol, which indicated that the HPA axis was activated during the long-term SARA challenge. This is consistent with limited, preliminary evidence that high concentrate diet (70% dry matter) adjustment to one-year-old Holstein heifers for 30 days increased blood total glucocorticoids from 7.9 to 14.8 ng/mL [37]. It's suggested that the increased concentration of glucocorticoids may be reflecting a metabolic adaptation to high concentrate feed [37]. In this study, hepatic GR protein expression was significantly down-regulated in liver of HC goats indicating a negative feedback from the higher level of plasma cortisol. As a primary and vital organ that is involved in the immune response and in nutrient metabolism, the liver controls the input of precursors of milk components to the mammary gland [24] [38]. These changes of HPA axis may be aimed on redistribution of energy utilization in specific organs, inhibiting or stimulating energy mobilization according to priority [26].

### The profile of mRNA expression in liver

In this study, microarray technology was used to investigate the hepatic gene expression profile and high-throughput data analysis was used to compare the differential expression of functional genes between HC and LC group. The Co-expression network of LC group shows in Figures S1, S2, S3, and S4. The activation of inflammatory and stress response in HC group was also confirmed from the GeneRelNet (Co-expression network) dataset. The results showed that the significant up-regulation (FC >2, p<0.05) of key-core gene regulated genes included CD1 (NM_001123002) and CD4 (NM_001129902) molecules, IL-2 (X55641.1) and corticotropin releasing hormone binding protein (CRHBP, NM_001009339) in liver of HC goats. These genes are important for immune response, cell adhesion, and antigen presentation. It's well known that CD4 molecule plays a key role for IL-2 produce [39] and CD1 protein is important for the innate and adaptive immune response [40]. Moreover, the significant up-regulation of 3-hydroxybutyrate dehydrogenase 1 (BDH1, NM_001034600.1) and Dipeptidase 1 (DPEP1, NM_001034472.1), and the down-regulation of glutathione peroxidase 2 (GPX2, NM_001163139.1) and aldehyde dehydrogenase 18A1 (ALDH18A1, NM_001046181.1) indicated the decrease of anti-oxidative capacity in liver of goat feeding high concentrate diet [41] [42] [43]. In a good agreement, feeding high concentrate diets enrich starch to dairy cow induced SARA disease and led to systemic inflammatory response due to the translocation of LPS from gastrointestinal tract into the peripheral circulation [10] [41] [42].

In addition to being an energy source, protein plays a functional role in many organs including the liver. The changes in protein metabolism are critical to maintaining cell size and directly impacts cell survive. Gene ontology analysis also identified 26 differential expression genes related to protein metabolism. Majority of these genes are involved in the biological processes of proteolysis, collagen catabolism, and purine nucleotide metabolism. Among these genes, two ATPase (NM_001083653.1, XM_001788345.1), branched chain amino-acid transaminase 1 (BCAT1, NM_001083644.1) and fascin homolog 3 (FSCN3, NM_001075543.2) were significantly up-regulated in HC goats, while RNA polymerase III (NM_001083750.1), and three kinases (XM_866451.4, NM_001109962.1, NM_001078008.1) were markedly down-regulated. These changes likely indicated the priority of protein catabolism in liver of HC goats compared to LC. It's reported that the elevation of plasma cortisol levels inhibits protein synthesis while increasing protein breakdown [43]. In this study, the relationship between increased plasma cortisol level and protein breakdown still warrants further investigation.

The priority of catabolism was also observed in lipids metabolism. Our results showed that the transcription of genes encoding three key enzymes acting in fatty acids β-oxidation [44], ACAA2, ACSL1 and HADH were up-regulated in liver of HC goats, which was consistent with the increase of NEFA level in blood. As a metabolic hormone, leptin plays vital roles on food intake, energy expenditure, and body weight mediated by leptin receptors situated in the hypothalamus [45]. Leptin has also been shown to have effects on whole body glucose and lipid metabolism [46]. In this study, the significant increase of leptin in HC goats paralleled the increase of NEFA, which indicates the stimulatory effect of leptin on lipids catabolism. With respect to glucose metabolism, key-core gene glycogen synthase (GYS2, NM_001192905.1) mRNA transcription was down-regulated in HC goats. In addition, Q-PCR results showed that mRNA expression of two important gluconeogenic genes hexokinase (HK) and pyruvate carboxylase (PC) genes was significantly down-regulated in liver of HC group. These data suggests the decrease of glucogen synthesis and gluconeogenesis in liver of HC group. However, this is contradictory to the increase of blood glucose concentrations, which was significantly elevated in HC group. This increase of blood glucose might be related to the production of lactose in these lactating animals [47]. Previous study showed that fatty acids have shown to produce an insulin-resistant state via cascade signaling pathway involving protein kinase C (PKC) and AKT phospholation [48]. Also, high levels of free fatty acids in the plasma have been shown to induce insulin resistance [49]. However, whether the increase of blood glucose in HC group is due to insulin-resistance is still unclear.

Taken as a whole, our data suggest that feeding HC diet to lactating goats for long period caused the activation of the inflammatory and stress response, and altered substrates metabolism in liver. Based on the data presented in this study, limited conclusions can be drawn from just measuring mRNA abundance because the post-transcription, protein translation, and the amount of active protein are dependent on many factors [50]. To our knowledge, it's the first time to report the changes of metabolism in liver after feeding HC diet to lactating ruminants, and the determination of wide-genome expression in liver will contribute to find the specific biochemical pathways.

### Conclusion

In summary, feeding a high-concentrate diet to lactating goats for the long-term will result in the depression of milk fat and milk protein. Activation of the stress response, the acute phase response stimulation and the inflammatory response may contribute to nutrient partitioning and re-distribution of energy in the liver, and ultimately lead to a decline in milk quality.

## Materials and Methods

### Ethics Statement

The experiment was conducted following the guidelines of Animal Ethics Committee at Nanjing Agricultural University, China. The study was approved by Animal Ethics of Nanjing Agriculture University. The sampling procedures complied with the the “Guidelines on Ethical Treatment of Experimental Animals” (2006) No. 398 set by the Ministry of Science and Technology, China and “the Regulation regarding the Management and Treatment of Experimental Animals” (2008) No.45 set by the Jiangsu Provincial People's Government.

### Animals

Eight health multiparous mid-locating goats with average initial BW 47±8 kg (mean ± SD) were housed in individual stalls in a standard animal feeding house at Nanjing Agricultural University (Nanjing, China). Animals were cannulated in rumen and were randomly allocated to two groups, one receiving diets with low concentrate (40% of dry matter) as the LC group (n = 4), and another receiving high concentrate diet (60% of dry matter) as the HC group (n = 4) for 9 weeks. Ingredient and nutrient composition of experimental diets were presented in Table 5. Goats were free access to fresh water throughout the experimental time.

**Table 5:** Nutrient composition and forge to concentrate ratio (F:C) of the TMR.

	Diet	
Items	LC	HC
Leymus chinensis	40	26.7
Alfalfa silage	20	13.3
Corn	22.99	23.24
Bran	0	20.77
Soybean meal	15	13.66
Limestone	0.65	1.43
CaHPO_4_	0.46	0
Premix^a^	0.5	0.5
NaCl	0.4	0.4
Total	100	100
Nutrient levels^b^		
ME (MJ/kg)	5.63	5.83
DCP %	9.9	10
NDF %	36.64	34.55
ADF %	24.74	20.35
EE %	2.87	3.21
NFC %	31.76	35
Ca %	0.8	0.9
P %	0.33	0.38
H20%	88.4	88.1

^a^ Amount of premix added, VA: 6000 IU/kg, VD: 2500 IU/kg, VE: 80 mg/kg, Cu: 6.25 mg/kg, Fe: 62.5 mg/kg, Zn: 62.5 mg/kg, Mn: 50 mg/kg, I: 0.25 mg/kg, Se: 0.125 mg/kg, Co: 0.125 mg/kg, Mo: 0.125 mg/kg.

^b^ Nutrient levels were calculated values.

LC, low concentrate; HC, high concentrate.

### Milk composition analysis

Fifty milliliter of milk were sampled every two weeks into the vials with potassium dichromate at 8:00 in the morning, and stored at the 4°C until milk fat, protein and lactose analysis conducted by the commercial company of Nanjing Weigang dairy industry Co., Ltd. The data were analyzed by Univariate using the General Linear. Models of SPSS 11.0 for Windows (StatSoft Inc, Tulsa, OK, USA).

### Rumen fluid collecting and measurements

Fifteen minutes prior to feed delivery and 1, 2, 3 and 5 h (or 1, 3, 5 h) after feed delivery on 2 consecutive days during 2, 4, 6 and 8 week of experimental period, 15 mL rumen fluids was collected with nylon bag and divided into 5 mL for pH measurement immediately with pH-meter. Another 10 mL were transferred into tubes and kept on ice and then centrifuged at 1,900×g at 4°C for 30 min. After that, the supernatant was collected and stored at −20°C until for LPS analysis. The concentration of LPS in rumen fluid was determined by a chromogenic endpoint assay (CE64406, Chinese Horseshoe Crab Reagent Manufactory Co., Ltd., Xiamen, China) with a minimum detection limit of 0.1 EU/mL. The procedures were performed according to the manufacture's instruction. The data of pH and LPS concentrations were analyzed by Univariate using the General Linear Models of SPASS 11.0 for windows (StatSoft Inc, Tulsa, OK, USA).

### Measurement of plasma biochemical parameters

At the end of experiment, plasma was sampled fifteen minutes prior to feed delivery using EDTA-containing vacuum tubes from jugular vein. Plasma leptin concentration was measured with radioimmunoassay (RIA) using commercial kits purchased from Shanghai Institute of Biological Products. The ranges of assay sensitivity were between 0.5 and 24 ng/mL. The inter- and intra-assay coefficients of variation were 5% and 10%, respectively. The concentration of NEFA (A042) and glucose was detected by commercial kits purchased from Nanjing Jiancheng Bioengineering institution, and the procedures were performed according to the manufacture's instruction. The concentrations of leptin and glucose were carried out in Nanjing General Hospital of Nanjing Military. Inflammatory cytokines such as IL-6 (RGB-60023G, Beijing Rigorbio Science Development Co., Ltd.), TNFα (RGB-60080G) and acute phase protein such as LBP (RGB-60266G), HP (H086-09, abcam), SAA (ab100635, abcam) were detected by ELISA kits. The concentration of LPS in plasma was determined by a chromogenic endpoint assay with diazo coupling reagent (CE80545, Chinese Horseshoe Crab Reagent Manufactory Co., Ltd., Xiamen, China) with a minimum detection limit of 0.01 EU/mL. The procedures were performed according to the manufacture's instruction. The data were analyzed by Independent-Samples T test using the Compare Means of SPASS 11.0 for Windows (StaSoft Inc, Tulsa, OK, USA).

### RNA extraction and real-time quantitative PCR

The liver tissues were sampled by a punch biopsy with a local anesthesia. Total RNA was extracted from each dairy goat using TRIZOL reagent (Takara, Dalian, China) according to the manufacturer's protocols. Total RNA concentration was then quantified by measuring the absorbance at 260 nm in a spectrophotometer (Eppendorf Biotechnology, Hamburg, Germany). Aliquots of RNA samples were subjected to electrophoresis with 1.4% agarose-formaldehyde gels stained with ethidium bromide to verify their integrity. Reverse transcription (RT) was performed using the total RNA (2 μg) in a final volume of 25 μL containing 1× RT-buffer, 100 U reverse transcriptase, 8 U RNase inhibitor (Promega, USA), 5.3 μmol/L random hexamer primers and 0.8 mmol/L dNTP (TaKaRa, Dalian, China). After incubation at 37°C for 1 h, the reaction was terminated by heating at 95°C for 5 min and quickly cooling on ice. Real-time PCR was performed in Mx3000P (Stratagene, La Jolla, CA, USA). Mock RT and No Template Controls (NTC) were set to monitor the possible contamination of genomic DNA both at RT and PCR. Melting curves were performed to insure a single specific PCR product for each gene. Two microliter of 16-fold dilution of RT product was used for PCR in a final volume of 25 μL containing 12.5 μL SYBR Green Real-time PCR Master Mix (TOYOBO Ltd., Shanghai, China) and 0.6–0.8 μM of each forward and reverse primers for target genes (as shown in Table 6). Goat β-actin mRNA was used as a reference gene for normalization purposes. The following PCR protocols were initial denaturation (1 min at 95°C), then a three-step amplification program (20 s at 95°C, 20–30 s at 60–64°C, 30 s at 72°C) was repeated 45 times. The method of 2^−ΔΔCt^ was used to analyze qPCR data with Independent-Samples T test using Compare means of SPSS 11.0 for Windows to statistical analysis. All samples were included in the same run of RT-PCR and repeated for at least 3 times. The primers were shown in Table 6. The data was analyzed by Independent-Samples T test using the Compare Means of SPASS 11.0 for Windows (StaSoft Inc, Tulsa, OK, USA).

**Table 6:** PCR primers for immunity and stress, lipid metabolism and carbohydrate metabolism.

Target genes	Genbank accession	PCR products (bp)	Primer sequences
β-actin	AF_481159	260	F:5′-CGGGATCCATCCTGCGTCTGGACCTG-3′
			R:5′-GGAATTCGGAAGGAAGGCTGGAAGAG-3′
ACAA2	XM_004020663.1	165	F:5′-TGTCTGCTGGCAAAGTCTCACC-3′
			R:5′-AACCAGAGCCACAGAGCCTGTT-3′
HADH	XM_004009637.1	197	F:5′-GAGTTTGTGGCGAAGACCCTGA-3′
			R:5′-GGCTTGTGATCTGCAAAGAGGAAG-3′
CPT1	Y18830.1	158	F:5′-GCTTTGATCGACACTTGTTTGCTC-3′
			R:5′-GCAAAGCAGCCGATGTTCACT-3′
CPT2	BC105423.1	157	F:5′-GCTTTGATCGACACTTGTTTGCTC-3′
			R:5′-GCAAAGCAGCCGATGTTCACT-3′
ACSL1	NM_001076085.1	191	F:5′-GCAACCCCAAAGGAGCAATG-3′
			R:5′-AGCAGCCTGATATCTCCTTGG-3′
PPARα	HM600810.1	243	F:5′-TAAAGCCAACCAAGATAACCC-3′
			R:5′-TCACCAAACAGCCGAAGA-3′
ATGL	GQ918145.1	180	F:5′-GGAGCTTATCCAGGCCAAT-3′
			R:5′-TGCGGGCAGATGTCACTC-3′
ACSS1	BC055008.1	242	F:5′-TCCTTGGCTGGGAGGATCAA-3′
			R:5′-TGTTGTCTGTCCTGTGAGCCA-3′
LPL	JQ670882.1	235	F:5′-TTCAGAGGCTATTACTGGAAATCC-3′
			R:5′-ATGTCAATCACAGCATTCATTCTACT-3′
SCD	AF325499.1	178	F:5′-TGCTGACAACTTATCTGGATGC-3′
			R:5′-AAGGAATCCTGCAAACAGCTA-3′
DGAT1	DQ380249.1	240	F:5′-TGCCTCAGACACTTCTACAAGCC-3′
			R:5′-GCCCGATGATGAGTGACAGC-3′
DGAT2	AJ519787.1	234	F:5′-CACTGGCTCCAGCATCCTCTC-3′
			R:5′-TTCTTGGGTGTGTTCCAGTCA-3′
FADS1	XM_004019593.1	187	F:5′-CCTTGCTGCCTCTATACTTCCA-3′
			R:5′-ACAAACCAGTTGCTTTCCAGGA-3′
FADS2	AY.850395.1	257	F:5′-AGAGCATCGCCTGGTTCACTA-3′
			R:5′-CCTTGTGGAAGACATTGGGTTT-3′
ACACA	NM_001009256	230	F:5′-CATGGAAATGTACGCGGACC-3′
			R:5′-GGTGGTAGATGGGAAGGAGGA-3′
FAS	XM_004013447.1	112	F:5′-TGCTCATTCACTCGGGTTCT-3′
			R:5′-AGGTATGCCCGCTTTTCG-3′
PPARγ	JN854246.1	238	F:5′-CATTTCTGCTCCGCACTAC-3′
			R:5′-TGGAACCCTGACGCTTT-3′
PC	NM_177946	187	F:5′-TCGCACCATGTATGTCATCCC-3′
			R:5′-AGGCTTTTTTAAAGGCAGAGGG-3′
FBP1	NM_001034447	106	F:5′-GCGGTCAAAGCCATCTCCAC-3′
			R:5′-CATCCAGCTTCTTCACTTGATCTCC-3′
G6Pase	EF062861	158	F:5′-AATGTCATGTTGTGGTTGGGATTCT-3′
			R:5′-GCATTGTAGATGCTCTGGATGTGG-3′
HK1	AM492192	139	F:5′-GCGGCTCTCTGATAAAACTCTGTTA-3′
			R:5′-TGAGCCATCGGGAATAGACCTTAC-3′
PYGL	AY827551	150	F:5′-GCCTTCCCAGATCAGGTTGC-3′
			R:5′-GTGGTTGGTGTAGGCGAAGGTC-3′
LEPR	NM_001009763	110	F:5′-GGAAGGAGTAGGGAAACCGAAGA-3′
			R:5′-TTGAGGAGGAGATTATTATTGGCAC-3′
GYS2	NM_001192905	131	F:5′-TTGGGCGGTATCTTTGTG-3′
			R:5′-AGCACAATGGACGGAAGC-3′
GSK3α	NM_001102192.1	250	F:5′-TGGCTTACACAGACATCAAA-3′
			R:5′-TCGGGCACATATTCCAGCAC-3′
GSK3β	NM_001101310.1	249	F:5′-AGACAAAGATGGCAGCAAGGTGAC-3′
			R:5′-ACGCAATCGGACTATGTTAC-3′
GLUT2	NM_001103222.1	268	F:5′-CATCCATCTTCCTCTTTGTCTG-3′
			F:5′-GATTTTCCTTTGGTTTCTGG-3′
IR	XM_590552.4	275	F:5′-GACGCAGGCCGGAGATGACCA-3′
			R:5′-GCTCCTGCCCGAAGACCGACTC-3′
SAA	AF540564.1	121	F:5′-CATCCTGCGTCTGGACCTGG-3′
			R:5′-TTCCTTGATGTCACGGACGATTT-3′
TLR4	JQ342090.1	195	F: 5′-GTTTCCACAAGAGCCGTAA-3′
			R: 5′- TGTTCAGAAGGCGATAGAGT-3′
MyD88	JQ308783.1	98	F:5′-ACAAGCCAATGAAGAAAGAG-3′
			R:5′-GAGGCGAGTCCAGAACC-3′
CD14	NM_001077209.1	239	F:5′-CCGTTCAGTGTATGGTTGCC-3′
			R:5′-TGCTTCGGGTCGGTGTT-3′
HP	XM_004015111.1	162	F:5′-TAATGCCCATCTGCCTAC-3′
			R:5′-CGCCCTCATAGTGTTTCA-3′
IL-1β	D63351.1	172	F: 5′-GAAGAGCTGCACCCAACA-3′
			R: 5′- CAGGTCATCATCACGGAAG-3′
TNF-α	AF276985.1	173	F: 5′-CAAGTAACAAGCCGGTAGCCC-3′
			R:5′-CCTGAAGAGGACCTGCGAGTAG-3′
LBP	XM_004014566.1	138	F: 5′-TGGAGCCAGGAAAGATAC-3′
			F: 5′-AGCCTTCTGCCAACTTAT-3′
CRP	NM_001144097.1	134	F: 5′-CTGGCTTGGGAGATTG-3′
			F: 5′-AGTGAGGGTAAGGGATT-3′

ACAA2, acetyl-coenzyme A acyltransferase 2; ACACA, acetyl-coenzyme A carboxylase alpha; ATGL, adipose triglyceride lipase; ACSL1, acyl-CoA synthetase long-chain family member 1; ACSS1, acyl-CoA synthetase short-chain family member 1; CD14, CD14 molecule; CPT1, carnitine palmityl transferase1; CPT2, carnitine palmityl transferase 2; CRP, C-reactive protein; DGAT1, diacylglycerol acyltransferase 1; DGAT2, diacylglycerol acyltransferase 2; FADS1, fatty acid desaturase 1; FADS2, fatty acid desaturase 2; FBP1, fructose-1,6-bisphosphatase 1; PPARα, peroxisome proliferator-activated receptor α; FAS, fatty acid synthase; G6Pase, glucose-6-phosphatase; GLUT2, solute carrier family 2 (facilitated glucose transporter), member 2; GYS2, glycogen synthase 2; GYSK3α, glycogen synthase kinase 3 alpha; GYSK3β, glycogen synthase kinase 3 beta; HADH, 3-hydroxyacyl-CoA dehydrogenases; HK1, hexokinase 1; HP, haptoglobin; IL1β; interleukin-1beta; IR, insulin receptor; LBP, lipopolysaccharide binding protein; LEPR, leptin receptor; LPL, lipoprotein lipase; MyD88, myd88; PC, pyruvate carboxylase; PPARγ, peroxisome proliferators-activated receptor γ; PYGL, liver glycogen phosphorylase; SAA, serum amyloid A protein; SCD, stearoyl-CoA desaturase; TLR4, toll-like receptor 4; TNFα, tumor necrosis factor alpha.

### Western blotting

Total protein was extracted from frozen liver tissue, and the protein concentration was determined by the BCA assay (Pierce, Rockford, IL, USA). Fifty micrograms of protein extract from each sample were subjected to electrophoresis on a 10% SDS-PAGE gel, and the separated proteins were transferred onto the nitrocellulose membranes (Bio Trace, Pall Co., USA). Western blot analysis for GR (sc-1004, Santa Cruz Biotechnology, 1∶500) is performed with primary antibodies and corresponding HRP-conjugated secondary antibodies. β-actin (KC-5A08, KangChen Bio-tech, China, 1∶5000) was used as a reference protein for normalization purposes in the Western blot analysis. Finally, the blot was washed and detected by enhanced chemiluminescence (ECL) using the LumiGlo substrate (Super Signal West Pico Trial Kit, Pierce, USA). ECL signals were recorded by an imaging system (Bio-Rad, USA) and analyzed with Quantity One software (Bio-Rad, USA). Values of GR protein was presented as fold change relative to the average value of LC group. The data was analyzed by Independent-Samples T test using the Compare Means of SPASS 11.0 for Windows (StaSoft Inc, Tulsa, OK, USA).

### mRNA Microarray Experiment

Transcriptome microarrays were performed for seven dairy goats (3 goats from the LC group and 4 goats from the HC group) for hepatic gene expression profiling. Because complete genome sequence data was not available, gene sequences from three animal species were employed to design the gene chip (goat, sheep, and *Bos Taurus*). Goat expressed sequence tag (EST) sequence (n = 13898), goat gene sequence (n = 720), sheep gene sequence (n = 2718), and *Bos Taurus* gene sequence (n>35000) transcripts were investigated using the microarray. There were 61012 probe IDs and 2-fold difference probe IDs (n = 2140). Reference sequences were cited from http://www.ncbi.nlm.nih.gov/gene/and EST database was from http://www.ncbi.nlm.nih.gov/nucest/. The FDR (False Discovery Rate) were calculated. Differentially expressed genes (DEGs) were selected with FDR <5% and FDR <10%. All data were MIAME compliant and have been deposited in GEO (accession number GPL17623 and GPL17624).

### GO analysis

GO analysis was applied to analyze the main function of the differential expression genes according to the Gene Ontology which is the key functional classification of NCBI, which can organize genes into hierarchical categories and uncover the gene regulatory network on the basis of biological process and molecular function [51]. Specifically, two-side Fisher's exact test and 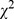 test were used to classify the GO category, and the false discovery rate (FDR) [52] was calculated to correct the P-value,the smaller the FDR, the small the error in judging the p-value. The FDR was defined as 
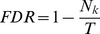
, where 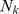 refers to the number of Fisher's test P-values less than 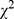 test P-values. We computed P-values for the GOs of all the differential genes. Enrichment provides a measure of the significance of the function: as the enrichment increases, the corresponding function is more specific, which helps us to find those GOs with more concrete function description in the experiment. Within the significant category, the enrichment Re was given by: 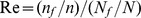 where “
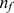
” is the number of flagged genes within the particular category, “
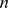
” is the total number of genes within the same category, “
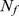
” is the number of flagged genes in the entire microarray, and “
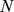
” is the total number of genes in the microarray [53].

### GeneRelNet (Co-expression network)

Gene co-expression Networks was established to identify the interactions among genes. Gene co-expression Networks were built according to the normalized signal intensity of specific expression genes [54]. For each pair of genes, we calculate the Pearson correlation and choose the significant correlation pairs with which to construct the network [55]. In a network analysis, degree centrality is the simplest and most important measures of a gene centrality within a network that determining the relative importance. Degree centrality is defined as the link numbers one node has to the other [56]. Moreover, to study a variety of properties of networks, k-cores were introduced in graph theory as a method of simplifying graph topology analysis. A k-core of a network is a subnetwork in which all nodes are connected to at least k other genes in the subnetwork. The Purpose of Network Structure Analysis is to locate core regulatory factors (genes). In one network, core regulatory factors connect most adjacent genes and have the biggest degrees. While considering different networks, Core regulatory factors were determined by the degree differences between two class samples [57].

## Supporting Information

Figure S1 **Co-expression network for immune and inflammatory responses in the liver of LC goats.**
(TIF)

Figure S2 **Co-expression network for lipid metabolism in the liver of LC goats.**
(TIF)

Figure S3 **Co-expression network for protein (or amino acid) metabolism in the liver of LC goats.**
(TIF)

Figure S4 **Co-expression network for carbohydrate metabolism in the liver of LC goats.**
(TIF)

## References

[1] Yang WZ, Beauchemin KA (2006) Effects of physically effective fiber on chewing activity and ruminal pH of dairy cows fed diets based on barley silage. J Dairy Sci 89: 217–228. 16357285 10.3168/jds.S0022-0302(06)72086-0

[2] Keunen JE, Plaizier JC, Kyriazakis L, Duffield TF, Widowski TM, et al (2002) Effects of a subacute ruminal acidosis model on the diet selection of dairy cows. J Dairy Sci 85: 3304–3313. 12512604 10.3168/jds.S0022-0302(02)74419-6

[3] Kleen JL, Hooijer GA, Rehage J, Noordhuizen JP (2003) Subacute ruminal acidosis (SARA): a review. J Vet Med A Physiol Pathol Clin Med 50: 406–414. 14633219 10.1046/j.1439-0442.2003.00569.x

[4] Plaizier JC, Krause DO, Gozho GN, McBride BW (2008) Subacute ruminal acidosis in dairy cows: the physiological causes, incidence and consequences. Vet J 176: 21–31. 18329918 10.1016/j.tvjl.2007.12.016

[5] Gozho GN, Plaizier JC, Krause DO, Kennedy AD, Wittenberg KM (2005) Subacute ruminal acidosis induces ruminal lipopolysaccharide endotoxin release and triggers an inflammatory response. J Dairy Sci 88: 1399–1403. 15778308 10.3168/jds.S0022-0302(05)72807-1

[6] Beauchemin KA, Yang WZ, Rode LM (2003) Effects of particle size of alfalfa-based dairy cow diets on chewing activity, ruminal fermentation, and milk production. J Dairy Sci 86: 630–643. 12647969 10.3168/jds.S0022-0302(03)73641-8

[7] Fairfield AM, Plaizier JC, Duffield TF, Lindinger MI, Bagg R, et al (2007) Effects of prepartum administration of a monensin controlled release capsule on rumen pH, feed intake, and milk production of transition dairy cows. J Dairy Sci 90: 937–945. 17235170 10.3168/jds.S0022-0302(07)71577-1

[8] Khafipour E, Krause DO, Plaizier JC (2009) Alfalfa pellet-induced subacute ruminal acidosis in dairy cows increases bacterial endotoxin in the rumen without causing inflammation. J Dairy Sci 92: 1712–1724. 19307653 10.3168/jds.2008-1656

[9] Li S, Khafipour E, Krause DO, Kroeker A, Rodriguez-Lecompte JC, et al (2012) Effects of subacute ruminal acidosis challenges on fermentation and endotoxins in the rumen and hindgut of dairy cows. J Dairy Sci 95: 294–303. 22192209 10.3168/jds.2011-4447

[10] Khafipour E, Krause DO, Plaizier JC (2009) A grain-based subacute ruminal acidosis challenge causes translocation of lipopolysaccharide and triggers inflammation. J Dairy Sci 92: 1060–1070. 19233799 10.3168/jds.2008-1389

[11] Nocek JE (1997) Bovine acidosis: implications on laminitis. J Dairy Sci 80: 1005–1028. 9178142 10.3168/jds.S0022-0302(97)76026-0

[12] Dong G, Liu S, Wu Y, Lei C, Zhou J, et al (2011) Diet-induced bacterial immunogens in the gastrointestinal tract of dairy cows: impacts on immunity and metabolism. Acta Vet Scand 53: 48. 21824438 10.1186/1751-0147-53-48 PMC3161887

[13] Gruys E, Toussaint MJ, Niewold TA, Koopmans SJ (2005) Acute phase reaction and acute phase proteins. J Zhejiang Univ Sci B 6: 1045–1056. 16252337 10.1631/jzus.2005.B1045 PMC1390650

[14] Bannerman DD, Paape MJ, Hare WR, Hope JC (2004) Characterization of the bovine innate immune response to intramammary infection with Klebsiella pneumoniae. J Dairy Sci 87: 2420–2432. 15328264 10.3168/jds.S0022-0302(04)73365-2

[15] Sohn MJ, Hur GM, Byun HS, Kim WG (2008) Cyclo(dehydrohistidyl-l-tryptophyl) inhibits nitric oxide production by preventing the dimerization of inducible nitric oxide synthase. Biochem Pharmacol 75: 923–930. 18061143 10.1016/j.bcp.2007.10.021

[16] Baumann H, Gauldie J (1994) The acute phase response. Immunol Today 15: 74–80. 7512342 10.1016/0167-5699(94)90137-6

[17] Heinrich PC, Castell JV, Andus T (1990) Interleukin-6 and the acute phase response. Biochem J 265: 621–636. 1689567 10.1042/bj2650621 PMC1133681

[18] Taguchi Y, Yamamoto M, Yamate T, Lin SC, Mocharla H, et al (1998) Interleukin-6-type cytokines stimulate mesenchymal progenitor differentiation toward the osteoblastic lineage. Proc Assoc Am Physicians 110: 559–574. 9824538

[19] Murata H, Shimada N, Yoshioka M (2004) Current research on acute phase proteins in veterinary diagnosis: an overview. Vet J 168: 28–40. 15158206 10.1016/S1090-0233(03)00119-9

[20] Heinrich PC, Behrmann I, Muller-Newen G, Schaper F, Graeve L (1998) Interleukin-6-type cytokine signalling through the gp130/Jak/STAT pathway. Biochem J 334 (Pt 2): 297–314. 10.1042/bj3340297 PMC1219691 9716487

[21] Zunszain PA, Anacker C, Cattaneo A, Carvalho LA, Pariante CM (2011) Glucocorticoids, cytokines and brain abnormalities in depression. Prog Neuropsychopharmacol Biol Psychiatry 35: 722–729. 20406665 10.1016/j.pnpbp.2010.04.011 PMC3513408

[22] Dentin R, Girard J, Postic C (2005) Carbohydrate responsive element binding protein (ChREBP) and sterol regulatory element binding protein-1c (SREBP-1c): two key regulators of glucose metabolism and lipid synthesis in liver. Biochimie 87: 81–86. 15733741 10.1016/j.biochi.2004.11.008

[23] Johnson RW (1997) Inhibition of growth by pro-inflammatory cytokines: an integrated view. J Anim Sci 75: 1244–1255. 9159271 10.2527/1997.7551244x

[24] Nukina H, Sudo N, Aiba Y, Oyama N, Koga Y, et al (2001) Restraint stress elevates the plasma interleukin-6 levels in germ-free mice. J Neuroimmunol 115: 46–52. 11282153 10.1016/s0165-5728(01)00260-0

[25] Alsemgeest SP, Lambooy IE, Wierenga HK, Dieleman SJ, Meerkerk B, et al (1995) Influence of physical stress on the plasma concentration of serum amyloid-A (SAA) and haptoglobin (Hp) in calves. Vet Q 17: 9–12. 7610559 10.1080/01652176.1995.9694521

[26] Grum DE, Drackley JK, Hansen LR, Cremin JD Jr (1996) Production, digestion, and hepatic lipid metabolism of dairy cows fed increased energy from fat or concentrate. J Dairy Sci 79: 1836–1849. 8923255 10.3168/jds.S0022-0302(96)76552-9

[27] Steele MA, Alzahal O, Walpole ME, McBride BW (2012) Short communication: grain-induced subacute ruminal acidosis is associated with the differential expression of insulin-like growth factor-binding proteins in rumen papillae of lactating dairy cattle. J Dairy Sci 95: 6072–6076. 22921628 10.3168/jds.2011-4864

[28] Garrett EF, Pereira MN, Nordlund KV, Armentano LE, Goodger WJ, et al (1999) Diagnostic methods for the detection of subacute ruminal acidosis in dairy cows. J Dairy Sci 82: 1170–1178. 10386303 10.3168/jds.S0022-0302(99)75340-3

[29] Oba M, Allen MS (2000) Effects of brown midrib 3 mutation in corn silage on productivity of dairy cows fed two concentrations of dietary neutral detergent fiber: 3. Digestibility and microbial efficiency. J Dairy Sci 83: 1350–1358. 10877401 10.3168/jds.S0022-0302(00)75002-8

[30] AlZahal O, Rustomo B, Odongo NE, Duffield TF, McBride BW (2007) Technical note: A system for continuous recording of ruminal pH in cattle. J Anim Sci 85: 213–217. 17179558 10.2527/jas.2006-095

[31] Duffield T, Plaizier JC, Fairfield A, Bagg R, Vessie G, et al (2004) Comparison of techniques for measurement of rumen pH in lactating dairy cows. J Dairy Sci 87: 59–66. 14765811 10.3168/jds.S0022-0302(04)73142-2

[32] Yang WZ, Beauchemin KA (2006) Effects of physically effective fiber on chewing activity and ruminal pH of dairy cows fed diets based on barley silage. J Dairy Sci 89: 217–228. 16357285 10.3168/jds.S0022-0302(06)72086-0

[33] Steele MA, Croom J, Kahler M, AlZahal O, Hook SE, et al (2011) Bovine rumen epithelium undergoes rapid structural adaptations during grain-induced subacute ruminal acidosis. Am J Physiol Regul Integr Comp Physiol 300: R1515–1523. 21451145 10.1152/ajpregu.00120.2010

[34] Minton JE (1994) Function of the hypothalamic-pituitary-adrenal axis and the sympathetic nervous system in models of acute stress in domestic farm animals. J Anim Sci 72: 1891–1898. 7928769 10.2527/1994.7271891x

[35] Kabaroff LC, Rodriguez A, Quinton M, Boermans H, Karrow NA (2006) Assessment of the ovine acute phase response and hepatic gene expression in response to Escherichia coli endotoxin. Vet Immunol Immunopathol 113: 113–124. 16806492 10.1016/j.vetimm.2006.04.003

[36] Karrow NA (2006) Activation of the hypothalamic-pituitary-adrenal axis and autonomic nervous system during inflammation and altered programming of the neuroendocrine-immune axis during fetal and neonatal development: lessons learned from the model inflammagen, lipopolysaccharide. Brain Behav Immun 20: 144–158. 16023324 10.1016/j.bbi.2005.05.003

[37] Mills SE, Jenny BF (1979) Effects of high concentrate feeding and fasting on plasma glucocorticoids in dairy heifers. J Anim Sci 48: 961–965. 479027 10.2527/jas1979.484961x

[38] Sander LE, Sackett SD, Dierssen U, Beraza N, Linke RP, et al (2010) Hepatic acute-phase proteins control innate immune responses during infection by promoting myeloid-derived suppressor cell function. J Exp Med 207: 1453–1464. 20530204 10.1084/jem.20091474 PMC2901069

[39] Zhu J, Yamane H, Paul WE (2010) Differentiation of effector CD4 T cell populations (*). Annu Rev Immunol 28: 445–489. 20192806 10.1146/annurev-immunol-030409-101212 PMC3502616

[40] Hussain MM, Rava P, Walsh M, Rana M, Iqbal J (2012) Multiple functions of microsomal triglyceride transfer protein. Nutr Metab (Lond) 9: 14. 22353470 10.1186/1743-7075-9-14 PMC3337244

[41] Emmanuel DG, Dunn SM, Ametaj BN (2008) Feeding high proportions of barley grain stimulates an inflammatory response in dairy cows. J Dairy Sci 91: 606–614. 18218747 10.3168/jds.2007-0256

[42] GN G, Plaizier Jc Fau – Krause DO, Krause Do Fau – Kennedy AD, Kennedy Ad Fau – Wittenberg KM, Wittenberg KM (2005) Subacute ruminal acidosis induces ruminal lipopolysaccharide endotoxin release and triggers an inflammatory response. 10.3168/jds.S0022-0302(05)72807-1 15778308

[43] Chen GL, Miller GM (2012) Advances in tryptophan hydroxylase-2 gene expression regulation: new insights into serotonin-stress interaction and clinical implications. Am J Med Genet B Neuropsychiatr Genet 159B: 152–171. 22241550 10.1002/ajmg.b.32023 PMC3587664

[44] Mashek DG, Li LO, Coleman RA (2007) Long-chain acyl-CoA synthetases and fatty acid channeling. Future Lipidol 2: 465–476. 20354580 10.2217/17460875.2.4.465 PMC2846691

[45] Stephens TW, Basinski M, Bristow PK, Bue-Valleskey JM, Burgett SG, et al (1995) The role of neuropeptide Y in the antiobesity action of the obese gene product. Nature 377: 530–532. 7566151 10.1038/377530a0

[46] Bryson JM, Phuyal JL, Swan V, Caterson ID (1999) Leptin has acute effects on glucose and lipid metabolism in both lean and gold thioglucose-obese mice. Am J Physiol 277: E417–422. 10484352 10.1152/ajpendo.1999.277.3.E417

[47] Xiao CT, Cant JP (2005) Relationship between glucose transport and metabolism in isolated bovine mammary epithelial cells. J Dairy Sci 88: 2794–2805. 16027193 10.3168/jds.S0022-0302(05)72959-3

[48] Gao Z, Zhang X, Zuberi A, Hwang D, Quon MJ, et al (2004) Inhibition of insulin sensitivity by free fatty acids requires activation of multiple serine kinases in 3T3-L1 adipocytes. Mol Endocrinol 18: 2024–2034. 15143153 10.1210/me.2003-0383

[49] Boden G, Homko C, Mozzoli M, Zhang M, Kresge K, et al (2007) Combined use of rosiglitazone and fenofibrate in patients with type 2 diabetes: prevention of fluid retention. Diabetes 56: 248–255. 17192489 10.2337/db06-0481

[50] Salisbury J, Hutchison KW, Wigglesworth K, Eppig JJ, Graber JH (2009) Probe-level analysis of expression microarrays characterizes isoform-specific degradation during mouse oocyte maturation. PLoS One 4: e7479. 19834616 10.1371/journal.pone.0007479 PMC2759528

[51] Ashburner M, Ball CA, Blake JA, Botstein D, Butler H, et al (2000) Gene ontology: tool for the unification of biology. The Gene Ontology Consortium. Nat Genet 25: 25–29. 10802651 10.1038/75556 PMC3037419

[52] Dupuy D, Bertin N, Hidalgo CA, Venkatesan K, Tu D, et al (2007) Genome-scale analysis of in vivo spatiotemporal promoter activity in Caenorhabditis elegans. Nat Biotechnol 25: 663–668. 17486083 10.1038/nbt1305

[53] Schlitt T, Palin K, Rung J, Dietmann S, Lappe M, et al (2003) From gene networks to gene function. Genome Res 13: 2568–2576. 14656964 10.1101/gr.1111403 PMC403798

[54] Pujana MA, Han JD, Starita LM, Stevens KN, Tewari M, et al (2007) Network modeling links breast cancer susceptibility and centrosome dysfunction. Nat Genet 39: 1338–1349. 17922014 10.1038/ng.2007.2

[55] Prieto C, Risueno A, Fontanillo C, De las Rivas J (2008) Human gene coexpression landscape: confident network derived from tissue transcriptomic profiles. PLoS One 3: e3911. 19081792 10.1371/journal.pone.0003911 PMC2597745

[56] Barabasi AL, Oltvai ZN (2004) Network biology: understanding the cell's functional organization. Nat Rev Genet 5: 101–113. 14735121 10.1038/nrg1272

[57] Carlson MR, Zhang B, Fang Z, Mischel PS, Horvath S, et al (2006) Gene connectivity, function, and sequence conservation: predictions from modular yeast co-expression networks. BMC Genomics 7: 40. 16515682 10.1186/1471-2164-7-40 PMC1413526

